# Use of Remote Monitoring by E-mail for Long-Term Management of the Classic Ketogenic Diet

**DOI:** 10.3390/nu12061833

**Published:** 2020-06-19

**Authors:** Cinzia Ferraris, Monica Guglielmetti, Elisa Tamagni, Claudia Trentani, Valentina De Giorgis, Ludovica Pasca, Costanza Varesio, Ottavia Eleonora Ferraro, Anna Tagliabue

**Affiliations:** 1 Human Nutrition and Eating Disorder Research Center, Department of Public Health, Experimental and Forensic Medicine, University of Pavia, Via Agostino Bassi, 21, 27100 Pavia, Italy; monica.guglielmetti@unipv.it (M.G.); elisatamagni@hotmail.it (E.T.); claudia.trentani@unipv.it (C.T.); anna.tagliabue@unipv.it (A.T.); 2 Department of Child Neurology and Psychiatry, IRCCS Mondino Foundation, Via Mondino, 2, 27100 Pavia, Italy; valentina.degiorgis@mondino.it (V.D.G.); ludovica.pasca01@universitadipavia.it (L.P.); costanza.varesio@gmail.com (C.V.); 3 Unit of Biostatistics and Clinical Epidemiology, Department of Public Health, Experimental and Forensic Medicine, University of Pavia, Via Forlanini, 2, 27100 Pavia, Italy; ottavia.ferraro@unipv.it

**Keywords:** ketogenic diet, drug-resistant epilepsy, glucose transporter type 1 deficiency syndrome, children, monitoring, home management, e-mail

## Abstract

The classic ketogenic diet (cKD) requires constant nutritional monitoring over time both to ensure its effectiveness and to reduce the likelihood of short- and long-term adverse effects. We retrospectively described the use of remote monitoring by e-mail during the first year of follow-up on cKD in 34 children (47% males; age range: 2−17 years) diagnosed with drug-resistant epilepsy (DRE; n = 14) or glucose transporter type 1 deficiency syndrome (GLUT1-DS; n = 20). All the e-mails were evaluated analyzing their frequency and content at 3, 6 and 12 months. Three families never sent e-mails. A median of 36.0 (IQR 23.0–64.0) e-mails per family were sent during the 12 follow-up months by the 31 patients. GLUT1-DS patients sent a greater number of e-mails than the DRE group (median 39.0 (IQR 25.5–56.5) vs. median 26.0 (IQR 19.0–65.0)). At the end of the follow-up period, a greater number of e-mails had been exchanged between the nutritional team and the families belonging to the group that increased its linear growth (median 83.5; IQR 48.0–102.0), compared to the other ones. Constant remote monitoring by e-mail could be a feasible and effective way for a better cKD management.

## 1. Introduction

Ketogenic dietary therapies (KDTs) are well-established, non-pharmacologic treatments used for children and adults with medication-refractory or drug-resistant epilepsy (DRE) [1], but they also represent the mainstay of treatment for glucose transporter type 1 deficiency syndrome (GLUT1-DS, OMIM 606777) [2]. Patients with DRE should receive KDTs for at least three months, after which they can be continued for years if symptoms subside (i.e., seizure reduction >50%) [3]. As far as subjects with GLUT1-DS are concerned, KDTs should be introduced with the expectation of a lifelong treatment [4]. KDTs require constant nutritional monitoring over time both to ensure their effectiveness and to reduce the likelihood of short- and long-term adverse effects [3]. The most frequently used ketogenic dietary therapy for epilepsy and GLUT1-DS is the classic ketogenic diet (cKD) which is a high-fat, very low carbohydrate, adequate-protein diet with a high ketogenic ratio (fat: carbohydrates + protein in weight) [4]. This is an isocaloric ketogenic diet (IKD) as defined by Trimboli et al. [5]. Despite its proven effectiveness, adherence to cKD is extremely demanding both for patients and for their families. Strict adherence to this dietary protocol can be very difficult; a patient starting it faces an abrupt switch of habits and must be properly trained to promote adherence to the treatment. It is not uncommon that patients experience serious problems in adhering to therapeutic menus that are often repetitive; they also complain about spending too much time cooking. cKD is particularly challenging in children, since, for these patients, the management of ketogenic ratio (KR) combines with increasing energy and nutrients requirements due to their growth. Food refusal and low compliance may impair adequate nutrition and cause growth delay in subgroups of children [6].

The recommendations of the International Ketogenic Diet Study Group about optimal clinical management reported that a multidisciplinary team—including pediatric neurologists, nutritionists, dietitians and pediatricians—should closely monitor patients to maximize the outcome of the therapy [3]. Phone or e-mail contact within the first month is advised; the child should be seen in clinic after 1 month as well, to ensure that the KDTs are being implemented correctly and to provide in-person early support. Afterward, children should be seen at 3, 6, 9, and then 12 months, with frequent e-mail or phone contact in-between. A child younger than 1 year of age should have even more frequent contact with the KDTs team [7]. Adjustments in KDTs or anti-epileptic drugs (AEDs) are typically performed for most children on KDTs, to reduce side effects and improve seizure control [7]. Those with a lower baseline seizure frequency and younger age of seizure onset were more likely to see additional benefit by fine-tuning modifications [8]. After 1 year on KDTs, visits can be spaced out to every 6 months with phone or e-mail contact in-between visits.

Based on our experience, careful long-term monitoring of dietary and clinical parameters is essential to support compliance, investigate possible side effects and promptly deal with them. There is a need for a solution that can facilitate more convenient and easy communication between families and physicians for a better therapy management and ensure proper patient follow-up, regardless of the distance.

To date, many experiments have confirmed the remarkable achievements of telehealth [9,10,11], which is increasingly being valued as a promising long-term management strategy for patients with chronic diseases. It can be an opportunity to remotely provide patients with consistent education, motivation to become engaged in their own self-care, and assistance in monitoring on a regular basis [12].

Recent studies have demonstrated the feasibility and efficacy of telemedicine in the field of neurology, also referred to as “teleneurology”, including in the care of patients with epilepsy [13,14,15].

Teleneurology is the use of modern communication technology to enable treatment when the doctor and patient are not present in the same place, and potentially not at the same time. The two main communication technologies used are videoconferencing equipment and e-mails, where the consultation is carried out without the patient being present and at a time convenient to the doctors involved [15]. The evidence gathered so far from the few faltering steps taken by teleneurology is that it can narrow the gap between patients with neurological disease and the doctors who are trained to look after them [15].

To our knowledge, the actual use of remote monitoring by e-mail has never been investigated before in patients on classic ketogenic diet treatment. The aim of this study is to retrospectively describe the use of remote monitoring by e-mail during the first year of follow up on cKD in 34 patients with GLUT1-DS and DRE. We wanted to evaluate the number and content of e-mails firstly in the whole group of patients and secondly to compare the results of subgroups of patients divided by diagnosis and growth pattern after 1 year on the diet.

## 2. Materials and Methods

### 2.1. Ethical Approval

The study protocol complied with the tenets of the Helsinki Declaration and was approved by the ethical committee of the Fondazione IRCCS Policlinico San Matteo of Pavia on 13 May 2019 (code number: 20190033749). All patients and/or caregivers provided their written informed consent.

### 2.2. Patients and Data Collection

All participants were consecutively enrolled at the Department of Child Neuropsychiatry of the IRCCS Casimiro Mondino Foundation (Pavia, Italy) between 2008 and 2017. Inclusion criteria were as follows: (1) age between 2 and 17; (2) diagnosis of DRE (defined as unresponsiveness to at least two anticonvulsant medications) or GLUT1-DS; and (3) treatment with cKD for at least 12 months. Children with severe organ failure, thyroid disorders, or requiring enteral and parenteral nutrition were excluded. Clinical data were extracted from a retrospective review of clinical records and entered onto an electronic database.

### 2.3. Ketogenic Diet

Using a standardized approach [16], the Human Nutrition Research Center keto-team implemented a non-fasting dietary protocol—which was uniformly applied to all of the study patients—characterized by gradual increases of the ketogenic ratio. The usual caloric intake as well as food intolerances and preferences were investigated using weekly food diaries. Energy prescriptions were tailored to each patient’s specific requirements (based on basal energy expenditures measured by indirect calorimetry and subsequently corrected for physical activity levels). cKD plans with increasing ketogenic ratios were adjusted at the individual level by an experienced dietician. Energy needs were reassessed every 3 months in infants and children up to 10 years old and every 6 months in adolescents. As far as macronutrient composition is concerned, a minimum of 0.8−1 g of proteins from animal sources (e.g., eggs, milk, meat, poultry, and fish) were supplied per kilogram of body weight. All of the study participants were prescribed sugar-free multivitamin and mineral supplements according to their age and sex.

Oral citrates were also administered to prevent the formation of kidney stones. All patients started at home on a 1:1 ketogenic diet, with ketogenic ratios subsequently increased to 2:1, 3:1, or 4:1 in order to achieve blood β-OHB levels ≥ 2.0 mmol/L. Family members were instructed to monitor blood β-OHB concentrations (twice per day every day during the induction phase and then once or twice a week) and report values by e-mail to the study investigators.

### 2.4. Follow-Up Visits and E-mail Monitoring

All participants underwent control visits 1 month after cKD initiation, then after 3, 6, 9, and 12 months, respectively. At follow-up visits, each patient underwent (1) measurements of fasting blood ketones, (2) assessments of compliance to the prescribed dietary regimen and supplement use, and (3) screening for potential adverse effects.

After the beginning of the diet and during the time intervals between control visits, parents or caregivers were asked to send e-mails in order to evaluate patients’ clinical response to the therapy and adjust ketogenic ratio prescription if needed. They were asked to report on diet compliance, beta-hydroxybutirate levels, side effects, dietary change requests or doubts about cKD management and supplement use.

All the e-mails exchanged between the patient’s family and the keto-team during the follow-up period after the beginning of the diet were analyzed. The total number, median frequency and e-mail contents were examined and collected in an Excel database. In particular, the total e-mail number and specifically those regarding dietary change requests (diet/menus modifications), ketonemia monitoring, adverse effects, supplements or drugs (information about the sugar content in supplements or changes with other products proposed by caregivers), blood chemistry, dietary info requests (requests related to specific foods, brands of products or how to divide meals if they are not all consumed during the day) and cKD suspension were analyzed and divided in 3-month periods (3, 6, 12), for the entire year of treatment.

The use of e-mail monitoring was analyzed in the group as a whole and in subgroups created according to diagnosis and growth pattern after one year on the diet [6].

### 2.5. Statistical Analysis

Quantitative variables were summarized by medians and interquartile ranges (IQRs), qualitative variables were summarized through percentages. The comparison across the three growth groups was performed using the non-parametric Kruskal–Wallis test, while to compare characteristics in different disease groups the Mann Whitney test was carried out. Associations between categorical variables were analyzed with the Chi-squared test or the Fisher’s exact test or the Fisher–Freeman–Halton’s exact test, as appropriate. The software used for the analysis was STATA/SE® for Windows, version 15 (StataCorp, College Station, TX, USA). Two-tailed p values < 0.05 were considered statistically significant.

## 3. Results

### 3.1. Patients

Table 1 shows the baseline characteristics of the entire study sample as well as separate values for patients with DRE and GLUT1-DS. Thirty-four children (16 male and 18 female) were included in the study. DRE was diagnosed in 14 children, whereas the remaining 20 had GLUT1-DS. In the entire sample, the median age at diagnosis was 7.5 (IQR 4.0–10.0) years and the age at the beginning of cKD ranged from 2 to 15 years (16 children were aged between 2 and 6, 10 between 7 and 10, and 8 between 11 and 17). Most of our patients (23/34) come from outside the Lombardy region.

Patients with GLUT1-DS were diagnosed at an older age (median 8.5 (IQR 5.0–10.0) years) than those with DRE (median 4.0 (IQR 1.0–7.5) years; *p* = 0.041) and began dietary treatment earlier after diagnosis (median interval 0.0 (IQR 0.0–1.0) years, vs. median 4.7 (IQR 3.5–6.5) years, respectively, *p* < 0.001)—an expected finding, since cKD is the only available treatment for GLUT1-DS.

There were significant differences in terms of anti-epileptic drugs (AEDs) and neurological impairment between the two groups. Specifically, patients with GLUT1-DS had minimal neurological impairment and none of them were taking AEDs. Ten patients with DRE were on multiple anti-epileptic drugs (AEDs) and their pharmacological treatment was not modified throughout the study. Most of the patients with DRE had severe neurological impairment.

The linear growth of the patients included in the present study has been described in a recent paper [5]. According to the 1-year change in height percentile, patients were classified in three subgroups: Group I (Increased), Group T (Tracking), Group D (Decreased). Details of the subgroups can be found in ref [5].

### 3.2. E-mail Monitoring Analysis in the Entire Sample

Three families never sent e-mails but maintained only phone contact, so they were excluded. The sample reduced to 31 patients.

As regards the whole sample (Table 2), a median of 36.0 (IQR 23.0–64.0) e-mails per person/family were sent during the 12 follow-up months by the 31 patients.

The content of the e-mails concerned primarily general or specific diet-related questions, a median of 16.0 e-mails (IQR 6.0–31.0) for patient were collected; families often had doubts about food choices (i.e., Is this food suitable for KD?) or how to manage hunger (i.e., Can I increase food consumption if it is not enough?).

A median of 6.0 e-mails related to both weekly or monthly registered ketonemia levels (IQR 4.0–17.0) and diet/menus modifications (IQR 2.0–13.0). We investigated possible correlation between blood β-OHB levels and total number of sent e-mails, but no significant differences emerged (data not shown).

A very low number of e-mails (median 1.0 (IQR 0.0–3.0)) concerned adverse effects (such as nausea, general tiredness, headache, vomiting) or supplements (like information about the sugar content of supplements or changes with other products proposed by caregivers).

No cKD suspension requests were sent during the entire year of treatment, except for one family.

E-mail response time by the nutritional team depended on the kind of request. “Simple” questions or topics (i.e., ketonemia levels, supplements or menu modifications) took a relatively brief time lapse, so we managed to give an answer within the same day. For more complex topics (i.e., adverse effects) that required team collaboration and decision-making, the time lapse could be more extended (from 2 days to one week).

### 3.3. E-mail Monitoring Analysis in Groups Basing on Pathologies

The study sample was composed of 13 subjects (42%) affected by DRE and 18 patients (58%) diagnosed with GLUT1-DS.

The use of e-mail monitoring was compared between these two groups of patients (Table 3) and it was noted that GLUT1-DS subjects sent a greater number of e-mails than the DRE group after 12 months (median 39.0 (IQR 25.5–56.5) vs. median 26.0 (IQR 19.0–65.0)), although no significant statistical difference was reported.

The amount of e-mails relating to dietary information differed between the two groups, although not in a statistically significant way (median DRE 8.0 (IQR 3.0–31.0) vs. GLUT1-DS median 18.0 (IQR 11.0–29.5)), while no request for diet suspension was e-mailed by either group.

The e-mails relating to adverse effects were greater in the DRE group after 12 months compared to the GLUT1-DS (median 3.0 (IQR 0.0–4.0) vs. median 0.0 (IQR 0.0–2.5)), although no significant statistical difference was reported.

### 3.4. E-mail Monitoring Analysis in Subgroups Based on the Growth Pattern

The use of e-mails was compared among the three subgroups based on the growth pattern (Table 4).

At the end of the follow-up period, a greater number of e-mails had been exchanged between the nutritional team and the families belonging to Group I (median 83.5 (IQR 48.0–102.0)) compared to Groups D and T (median 40.0 (IQR 21.0–65.0) and 30.0 (IQR 21.0–49.0), respectively), although no significant statistical difference was found.

This trend was found in all e-mail subgroups, but especially in the number of e-mails requiring information about diet management: a median of 28.5 e-mails (IQR 18.0–49.5) in Group I, 18.0 e-mails (IQR 8.0–34.0) in Group D and 11.0 e-mails (IQR 3.5–26.5) in Group T.

All groups sent a substantial number of e-mails about dietary change requests (menu modification) with a median of 19.5 e-mails (IQR 11.5–24.5) in Group I, 3.0 e-mails (IQR 0.0–6.0) and 5.5 e-mails (IQR 2.5–10.5) in Group D and Group T, respectively.

The e-mails relating to the sending of ketonemia values were greater in Group I, although no significant statistical difference was found. In particular, a median of 19.0 e-mails (IQR 9.0–33.5) was found in Group I, 6.0 e-mails (IQR 2.5–9.0), 6.0 e-mails (IQR 3.0–26.0) in Group D and Group T, respectively.

## 4. Discussion

In this study, we retrospectively analyzed the use of remote cKDs monitoring by e-mail during the first year of follow-up in 34 patients with GLUT1-DS and DRE.

Three families did not keep e-mail contact during the study period, instead they maintained only phone contact, so they were excluded and the final sample was composed of 31 families.

All the exchanged e-mails between the patient’s family and the nutritional team were examined, both in terms of frequency and content, at 3, 6 and 12 months after cKD initiation.

Our data showed that, in each period considered, GLUT1-DS families sent a greater number of e-mails compared to the DRE group. Specifically, in the first three months of diet, the GLUT1-DS group asked for diet modifications and dietary information more frequently than DRE patients. This trend could be explained by the fact that KD is actually the only therapy currently available for GLUT1-DS patients, who may be more motivated to a strict dietary adherence. Another explanation could be that GLUT1-DS patients had a minimal neurological impairment and a higher median age, so they could be more involved in choosing their menu. During the follow-up period, DRE patients’ caregivers sent a greater number of e-mails referring to adverse effects such as nausea, general tiredness, and vomiting. For those patients, the ketogenic diet is actually an alternative therapy, so it is possible that families tend to emphasize the onset or persistence of side effects. Another important factor to take into account is that DRE patients had severe neurological and clinical impairment that could have negatively influenced diet tolerability.

During the first 3 months, phone or e-mail contacts between patient/caregiver and the team are surely more frequent, since this is the induction period, during which greater support is needed in order to try to solve—in the shortest time possible—any difficulties or doubts regarding diet management. Moreover, during the first period, ketonemia levels and clinical response to the treatment are often inconstant and require a stricter contact with the family.

From the nutritional point of view, the most relevant topics expressed in the e-mails were dietary changes and info requests, ketonemia monitoring, reported adverse effects and multivitamin–mineral supplements information.

Lack of palatability of ketogenic food, as well as qualitative and quantitative restrictions in meal preparation, can be burdensome. Each ingredient needs to be weighed per gram, resulting in a long time spent for meal preparation; the overall treatment should be continuously monitored and tailored to the individual patient. A great aid to families is given by applications available for smartphones or software for personal computers, through which an appropriate count of nutrients can be easily calculated for each meal and recipes approved by dieticians can be shared. Adherence to cKD implies important limitations in participation in daily social activities, not only for patients but also for their families [17]. Despite these difficulties, our experience suggests that in the specific set of GLUT1-DS patients, compliance has proven to be better than in other conditions for which cKD is indicated [18], likely because of the efficacy of the treatment on symptoms.

In our previous study, we speculated that our promising results on growth may depend on an accurate assessment of energy needs (obtained by repeated measurements of resting energy expenditure and a thorough assessment of daily physical activity levels), an adequate protein-to-energy ratio according to WHO recommendations, as well as the continuing support offered to caregivers involved in the management of KD [6]. In the three growth subgroups analyzed, we observed that at 3, 6 and 12 months the total number of e-mails sent by Group I was more than doubled compared to Group D and almost three times the number sent by Group T. Furthermore, considering e-mail classification (dietary change requests, ketonemia monitoring, adverse effects, supplements and diet information requests) we noticed that the subgroup who experienced an increase in growth (Group I) actually had a more frequent e-mail contact with the nutritional team.

On this basis, we can hypothesize that constant monitoring during the first year of ketogenic diet could result in a greater adherence to the treatment.

The hypothesis that e-mail monitoring could be effective in ketogenic diet management was investigated only in one previous study conducted by Cervenka C.M. and colleagues a few years ago [13]. Although he considered a different ketogenic protocol known as Modified Atkins Diet (MAD) in epileptic adult patients, in his study he observed that e-mail management could be feasible and effective. More than 60% of the 22 participants who started MAD treatment completed the first three months of the study. Furthermore, 40% and 27% of the subjects obtained a >50% seizure reduction after 1 and 3 months of treatment, respectively. Similar results are described by Kossoff E.H. et al. [19], who studied 30 adult patients treated with MAD in an office setting with direct dietitian assistance. In that study, 47% had >50% reduction in seizure frequency at 1 month and 33% at 3 months. Side effects reported during e-mail MAD administration were also similar to those reported in prior studies and no serious or irreversible side effects were reported [20,21].

As reported in recommendations of the International Ketogenic Diet Study Group about optimal clinical management, phone or e-mail contact within the first month is advised [3]. Constant remote monitoring by e-mail could be a feasible and effective way for a better ketogenic diet management, especially in those patients who live far from the reference care center.

E-mail monitoring has a number of distinct advantages; for example, it can be read and accessed from different sites, so that the dietician or the physician can be in contact with the patient both from the hospital and from home/wherever he/she is and rapidly answer patients’ requests, if necessary. Using a structured pro forma to record the history and the basic neurological examination, it is possible to triage referrals into those patients who can be reassured, those who require straightforward investigations and those who need to be seen. In addition, this system enables a much better two-way communication between hospital specialists and other colleagues [12].

Another advantage could be that, in this way, the patient is monitored and rapidly helped by the nutritional team. In fact, e-mail contact allows for an easier communication with the dietician (that could also be on a daily basis) and to make any changes to the menus based on patients’ tastes and/or dietary prescription, increasing cKD adherence.

The caveats of our investigation include its retrospective design, the limited sample size and follow-up period, and the inability to detect telephone contacts that integrate monitoring. Moreover, the lack of a control group did not permit an investigation of the effects on compliance or efficacy.

These limitations notwithstanding, our study describes for the first time the use of remote monitoring during ketogenic diet treatment in DRE and GLUT1-DS patients.

Constant monitoring via email use by families, especially during the first months of the ketogenic diet, could in our experience actually lead to greater compliance by the patient and consequently have a positive effect on the normal growth process. Further studies are needed on the use of this type of remote follow-up, taking into account the cost/benefit ratio of the inclusion in the routine therapeutic process. Moreover, it would be useful and desirable to conduct future prospective case–control studies to evaluate the effect of monitoring via e-mail on adherence to the ketogenic diet.

## 5. Conclusions

Constant remote monitoring by e-mail could be a feasible and effective way for a better KDTs management, especially in those patients who live far from the reference care center. It seems to be very useful in the first 12 months of therapy, to support and help families facing for the first time all the aspects of cKD treatment.

## Figures and Tables

**Table 1:** Baseline characteristic of the entire sample and of drug-resistant epilepsy (DRE) and glucose transporter type 1 deficiency syndrome (GLUT1-DS) patients.

	ENTIRE SAMPLE	DRE	GLUT1-DS	*P*
	*n* = 34	*n* = 14	*n* = 20	
Sex (male/female)	16/18	7/7	9/11	>0.90
Age at diagnosis (years)	7.5 (4.0–10.0)	4.0 (1.0–7.5)	8.5 (5.0–10.0)	**0.041**
Age at ketogenic diet initiation (years)	8.0 (3.0–10.0)	4.0 (2.0–10.0)	9.0 (5.0–10.5)	0.310
Interval time between diagnosis and ketogenic diet initiation (years)	0.5 (0–4.2)	4.7 (3.5–6.5)	0.0 (0.0–1.0)	**<0.001**
Puberty (yes/no)	3/34	1/13	2/18	>0.90
Number of AEDs				**<0.001**
None	21/34	1/14	20/20	
1	3/34	3/14	0/20	
>1	10/34	10/14	0/20	
Severe neurological impairment				**<0.001**
None	11/34	1/14	10/20	
Mild	8/34	2/14	6/20	
Moderate	3/34	0/14	3/20	
Severe	12/34	11/14	1/20	

Data are reported as counts, medians, and interquartile ranges, as appropriate. Abbreviations: DRE, drug resistant epilepsy; GLUT1-DS, glucose transporter type 1 deficiency syndrome; AEDs, anti-epileptic drugs. Significant *p* values are marked in bold (*p* < 0.05) for comparison between DRE and GLUT1-DS patients.

**Table 2:** E-mail monitoring in total sample.

	3 Months	6 Months	12 Months
Total e-mails (*n*):	14.0 (6.0–33.0)	23.0 (11.0–46.0)	36.0 (23.0–64.0)
Dietary change requests (*n*):	2.0 (0.0–5.0)	3.0 (1.0–10.0)	6.0 (2.0–13.0)
Ketonemia (*n*):	4.0 (0.0–7.0)	5.0 (2.0–9.0)	6.0 (4.0–17.0)
Adverse effects (*n*):	0.0 (0.0–1.0)	0.0 (0.0–2.0)	1.0 (0.0–3.0)
Supplements (*n*):	1.0 (0.0–2.0)	1.0 (0.0–3.0)	1.0 (0.0–3.0)
Dietary info requests (*n*):	6.0 (1.0–14.0)	12.0 (2.0–18.0)	16.0 (6.0–31.0)
KD suspension (*n*):	0.0 (0.0–0.0)	0.0 (0.0–0.0)	0.0 (0.0–0.0)

Data are reported as medians and interquartile ranges. “Total e-mails” refers to the number of sent e-mails from the whole sample (*n* = 31 patients) at 3, 6, 12 months, respectively. There were no significant differences between 3, 6 and 12 months in each variable. Abbreviation: KD = ketogenic diet.

**Table 3:** E-mail monitoring in DRE and GLUT1–DS patients.

	DRE (*N* = 13)			GLUT1-DS (*N* = 18)		
	3 Months	6 Months	12 Months	3 Months	6 Months	12 Months
Total e-mails (*n*):	11.0 (4.0–30.0)	21.0 (11.0–38.0)	26.0 (19.0–65.0)	18.5 (6.0–33.0)	24.0 (13.5–46.0)	39.0 (25.5–56.5)
Dietary change requests (*n*):	2.0 (2.0–4.0)	2.0 (2.0–7.0)	5.0 (3.0–9.0)	3.5 (0.0–9.0)	4.5 (1.0–16.0)	6.0 (2.0–17.5)
Ketonemia (*n*):	4.0 (1.0–7.0)	5.0 (3.0–17.0)	9.0 (6.0–25.0)	3.5 (0.0–6.5)	4.0 (1.0–8.0)	5.5 (2.0–11.5)
Adverse effects (*n*):	0.0 (0.0–1.0)	0.0 (0.0–3.0)	3.0 (0.0–4.0)	0.0 (0.0–1.0)	0.0 (0.0–1.5)	0.0 (0.0–2.5)
Supplements (*n*):	1.0 (0.0–2.0)	1.0 (1.0–3.0)	1.0 (0.0–3.0)	0.0 (0.0–2.0)	1.0 (0.0–3.0)	1.5 (0.0–5.5)
Dietary info requests (*n*):	6.0 (0.0–16.0)	8.0 (2.0–22.0)	8.0 (3.0–31.0)	8.0 (1.0–13.0)	12.5 (4.4–17.5)	18.0 (11.0–29.5)
KD suspension (*n*):	0.0 (0.0–0.0)	0.0 (0.0–0.0)	0.0 (0.0–0.0)	0.0 (0.0–0.0)	0.0 (0.0–0.0)	0.0 (0.0–0.0)

Data are reported as medians and interquartile ranges. “Total e-mails” refers to the number of sent e-mails from epileptic (*n* = 13 patients) and GLUT1-DS (*n* = 18 patients) families at 3, 6, 12 months, respectively. There were no significant differences between 3, 6 and 12 months in each study group. Abbreviations: DRE = drug-resistant epilepsy, GLUT1-DS = glucose transporter type 1 deficiency syndrome, KD = ketogenic diet.

**Table 4:** E-mail monitoring in the three different growth subgroups.

	3 Months			6 Months			12 Months		
	Group I *N* = 5	Group T *N* = 20	Group D *N* = 6	Group I *N* = 5	Group T *N* = 20	Group D *N* = 6	Group I *N* = 5	Group T *N* = 20	Group D *N* = 6
Total e-mails (*n*):	37.5 (18.0–60.0)	10.0 (6.0–24.5)	28.0 (0.0–35.0)	55.5 (29.5–77.0)	19.5 (10.0–33.5)	31.0 (16.0–46.0)	83.5 (48.0–102.0)	30.0 (21.0–49.0)	40.0 (21.0–65.0)
Dietary change requests (*n*):	11.0 (5.0–17.5)	2.5 (0.5–4.5)	2.0 (0.0–2.0)	15.5 (9.5–19.5)	3.5 (1.0–8.0)	2.0 (0.0–3.0)	19.5 (11.5–24.5)	5.5 (2.5–10.5)	3.0 (0.0–6.0)
Ketonemia (*n*):	6.0 (4.5–10.5)	2.5 (0.0–5.0)	3.0 (0.0–11.0)	12.0 (5.5–20.5)	4.5 (0.5–6.5)	4.0 (3.0–21.0)	19.0 (9.0–33.5)	6.0 (2.5–9.0)	6.0 (3.0–26.0)
Adverse effects (*n*):	0.5 (0.0–1.0)	0.0 (0.0–0.5)	0.0 (0.0–2.0)	1.0 (0.0–3.5)	0.0 (0.0–2.0)	1.0 (0.0–3.0)	3.0 (1.0–6.0)	0.0 (0.0–3.0)	2.0 (1.0–4.0)
Supplements (*n*):	4.0 (2.0–5.5)	0.5 (0.0–1.0)	0.0 (0.0–1.0)	4.0 (2.0–5.5)	1.0 (0.0–2.0)	1.0 (0.0–3.0)	4.5 (2.0–7.0)	1.0 (0.0–2.0)	3.0 (1.0–3.0)
Dietary info requests (*n*):	18.0 (8.5–35.5)	5.0 (0.5- 10.0)	11.0 (0.0–16.0)	23.0 (12.5–42.5)	8.0 (2.0–15.0)	15.0 (7.0–22.0)	28.5 (18.0–33.5)	11.0 (3.5–26.5)	18.0 (8.0–34.0)
KD suspension (*n*):	0.0 (0.0–0.0)	0.0 (0.0–0.0)	0.0 (0.0–0.0)	0.0 (0.0–0.0)	0.0 (0.0–0.0)	0.0 (0.0–0.0)	0.0 (0.0–0.5)	0.0 (0.0–0.0)	0.0 (0.0–0.0)

Data are reported as medians and interquartile ranges. Each field relates to the total number of e-mails sent from patients with increased (*n* = 5 patients), tracking (*n* = 20 patients) and decreased (*n* = 6 patients) growth after 1 year of ketogenic treatment at 3, 6, 12 months, respectively. There were no significant differences between 3, 6 and 12 months in each study group. Abbreviations: Group T = Tracking group, Group I = Increased group, Group D = Decreased group, KD = ketogenic diet.
